# Simple Methods for Evaluating Acid Permeation and Biofilm Formation Behaviors on Polysiloxane Films

**DOI:** 10.3390/ma15062272

**Published:** 2022-03-19

**Authors:** Nobumitsu Hirai, Masaya Horii, Takeshi Kogo, Akiko Ogawa, Daisuke Kuroda, Hideyuki Kanematsu, Junji Nakata, Shigeru Katsuyama

**Affiliations:** 1 Department of Chemistry and Biochemistry, National Institute of Technology (KOSEN), Suzuka College, Shiroko-Cho, Suzuka 510-0294, Japan; ogawa@chem.suzuka-ct.ac.jp; 2 Advanced Engineering Course of Science and Technology for Innovation, National Institute of Technology (KOSEN), Suzuka College, Shiroko-Cho, Suzuka 510-0294, Japan; r03a22@ed.cc.suzuka-ct.ac.jp; 3 Department of Materials Science and Engineering, National Institute of Technology (KOSEN), Suzuka College, Shiroko-Cho, Suzuka 510-0294, Japan; kougo@mse.suzuka-ct.ac.jp (T.K.); daisuke@mse.suzuka-ct.ac.jp (D.K.); 4 Joint Research Center between Academia and Industries, National Institute of Technology (KOSEN), Suzuka College, Shiroko-Cho, Suzuka 510-0294, Japan; kanemats@mse.suzuka-ct.ac.jp; 5 Division of Materials and Manufacturing Science, Osaka University, Yamadaoka, Suita 565-0871, Japan; nakata@mat.eng.osaka-u.ac.jp (J.N.); katsuyama@mat.eng.osaka-u.ac.jp (S.K.)

**Keywords:** silane coating, silane compound, biofilm formation, acid permeation, sewerage systems, sewage, contact angle, adhesion work, wettability

## Abstract

The sulfuric acid permeation and biofilm formation behaviors of polysiloxane films have been investigated, and simple methods for evaluating the sulfuric acid permeation and biofilm formation behaviors have been proposed in this paper. The polysiloxane films used in these experiments were practically impermeable to the aqueous sulfuric acid solution, and the amount of biofilm formation varied depending on the composition of the films. Further, the amount of sulfuric acid permeation can be estimated by measuring the polarization curves of polysiloxane films with different thicknesses formed on iron electrodes. By measuring the adhesion work of pure water and simulated biofilm droplets on polysiloxane films of different compositions, we can estimate the resistance of biofilm formation on the polysiloxane films.

## 1. Introduction

Since the 1970s, Japan’s sewerage systems have grown remarkably, and the total length of sewerage culverts nationwide as of the end of the fiscal year 2019 was approximately 480,000 km. In 2019, sewerage culverts that have exceeded the standard service life of 50 years accounted for approximately 22,000 km (5% of the total length), 76,000 km (16% of the total length) 10 years later, and 170,000 km (35% of the total length) 20 years later [1]. The incidence of road cave-ins increases 30 years after the installation of sewage pipes [2], and the number of road cave-ins caused by pipeline facilities in Japan was approximately 2900 per year at the end of the fiscal year 2019. Road cave-ins caused by sewage pipes not only interfere with the sewerage system, such as sewage collection and drainage, but also have a major impact on human life and road traffic if large-scale culvert damage occurs. As a result, evaluating the condition of buried sewage pipes through various methods, such as in-drain surveys, and reconstructing or repairing them to prevent damage to old sewage pipes is important.

The formation of a coating on the inner walls of sewers using a sealant is one method for preventive sewer repair, preventing road cave-ins [3,4]. Preventing sulfuric acid penetration is a requirement for maintaining the performance of the coating films. Concrete sewage walls react with sulfuric acid to form gypsum dihydrate and ettringite, which drastically reduce the strength of the concrete walls. Therefore, the coating film must be impervious to sulfuric acid to prevent corrosion by sulfuric acid solution.

In addition to corrosion prevention, biofilm (sludge, dirt) on the inner walls is desirable [5,6]. When sulfate-reducing bacteria are present in the biofilm, they convert sulfuric acid into hydrogen sulfide, which induces hydrogen sulfide-induced metal corrosion (e.g., corrosion of manholes) in the absence of oxygen, in addition to the toxicity of hydrogen sulfide. Because of their ability to absorb a wide range of inorganic ions, biofilms can also cause scale. The scale is the precipitation of inorganic salts, such as calcium carbonate, calcium sulfate, and silica precipitated in water on the inner wall. Therefore, biofilm prevention performance is sought in the coating film for sewer interior walls.

As previously stated, the coating film for the inner wall must be capable of preventing sulfuric acid penetration and biofilm formation. However, because of the need to handle bacteria, the evaluation of sulfuric acid penetration prevention performance requires long-term experiments in a controlled environment, whereas the evaluation of biofilm prevention performance requires an appropriate experimental environment and experimental equipment. Therefore, simple test methods that may be used for screening are required.

Based on the foregoing, this study proposes simple methods for evaluating sulfuric acid penetration prevention performance and biofilm prevention performance that can be applied to coating films for sewer interior walls. Using the proposed methods, the sulfuric acid penetration prevention performance and the biofilm prevention performance can be evaluated in a short time and without the need for bacteria, respectively.

## 2. Materials and Methods

### 2.1. Polysiloxane Film

Polysiloxane films containing various components were applied to mortar, carbon steel (SS400), and glass substrates. The films used were of the alkoxysilane compounds, called “Permeate” [7] (D&D, Yokkaichi, Japan), and owing to their low viscosity (61.5 mPa·s), they easily permeated the pores of coatings. After interpenetrating pores and coating substrates, the films react with the moisture in the ambient atmosphere and harden, owing to the formation of inorganic polymers.

### 2.2. Immersion Experiment of Polysiloxane Film in Sulfuric Acid Solution

A sample of 200 µm-thick polysiloxane film formed on the mortar was immersed in a 5% sulfuric acid aqueous solution for 120 days. Following that, the sample was broken to expose the film’s cross-section, the broken sample was embedded in resin, and the sample’s surface was dry-polished with emery paper. Furthermore, the sample’s surface was coated with osmium tetroxide to make it conductive before being observed with an electron probe microanalyzer (EPMA). The EPMA used was JXA-8800 (JEOL, Tokyo, Japan).

### 2.3. Polarization Experiment of Polysiloxane Film Formed on Carbon Steel

On carbon steel (SS400) plates, samples of polysiloxane film with thicknesses 0, 15, and 30 µm were formed. The sample was then placed into an electrochemical cell. A platinum wire served as the counter electrode and a mercury/mercury sulfate electrode as the reference electrode. The electric potential shown in this paper was based on the mercury/mercury sulfate electrode. The electrolyte was a 5% solution of sulfuric acid. The potential was held at −1.1 V for 10 min after the electrolyte was introduced into the electrochemical cell. Then, the cell was held at rest potential for 10 min without energizing. After that, the potential was scanned from the resting potential to 0.8 V at a rate of 20 mV/min, and the corrosion current of the sample was measured. The corrosion current in the active state ranged from −0.6 to −0.4 V, allowing for a simple evaluation of sulfuric acid permeation through the films. The polarization curve was performed using the method described in the Japanese Industrial Standards [8] for measuring the anodic polarization curve of stainless steel.

### 2.4. Biofilm Formation Experiment on Polysiloxane Film

The amount of real biofilm produced by *Aliivibrio fischeri* [9] (JCM18803, RIKEN BioResource Center (BRC), Tsukuba, Japan) was investigated as follows: *Aliivibrio fischeri* was cultivated in Marine Broth at 22 °C for two days. Following that, the cultivated media were diluted fourfold with phosphate-buffered saline, and glass plates coated with polysiloxane films of eight compositions were placed on them at 22 °C for two days to form the biofilm. The polysiloxane films evaluated in this study were prepared using oligomer B and oligomer A, the latter of which contains more phenyl groups. Experiments were conducted on eight compositions of oligomer B/oligomer A ratios ranging from 100/0 to 30/70 in 10 increments. Finally, the plates were dipped in 0.1% crystal violet aqueous solution for biofilm staining, the stained biofilm was dissolved in pure water, and the absorbance was measured three times on each substrate using an ultraviolet (UV)–visible (vis) spectrophotometer at a wavelength of 580.0 nm (UV-1800, Shimadzu, Kyoto, Japan). The Student’s t-test was used to assess considerable differences between substrates of different compositions. Statistical significance was evaluated at *p* < 0.05.

### 2.5. Wettability Experiment of Pure Water and Simulated Biofilm Droplet on Polysiloxane Film

The following experiments were conducted with a DMo-501 contact angle meter (Kyowa Surface Science, Niiza, Japan). Glass plates were coated with polysiloxane films of eight compositions, which were the same as for the biofilm formation experiment. On each substrate, the contact angle was measured four times using 2 µL of pure water droplets or 1% alginate aqueous solution droplets. Alginate is the main component of *Pseudomonas aeruginosa* biofilm [10] and is one of the biofilm model substances [11]. The different adhesion properties of pure water droplets or 1% alginate aqueous solution droplets on each substrate were used to estimate the simple evaluation of biofilm formation on the films. The Student’s t-test was used to assess considerable differences between substrates of different compositions. Statistical significance was evaluated at *p* < 0.05, *p* < 0.01, and *p* < 0.001.

## 3. Results and Discussion

### 3.1. Immersion Experiment of Polysiloxane Film in Sulfuric Acid Solution

**Figure 1:**
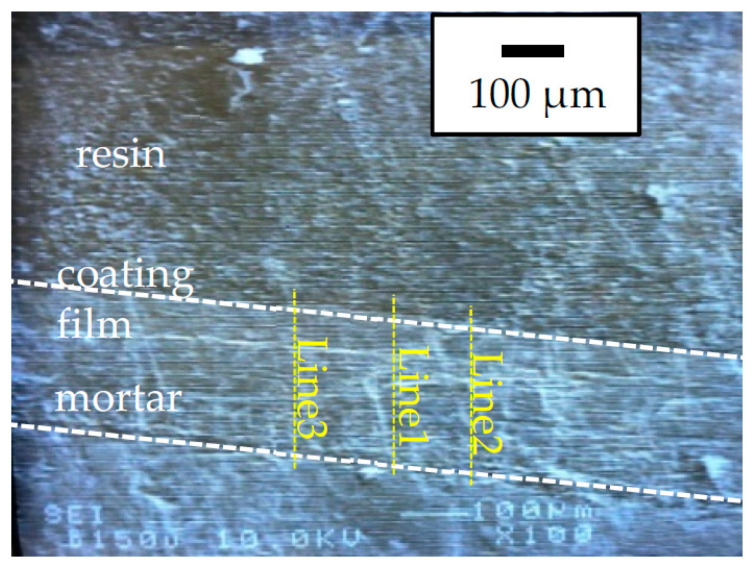
Microscopic image of the sample with three lines describing where EPMA line analysis was performed.

Figure 1 shows a microscopic image of the sample with three lines describing how the EPMA line analysis was performed. The resin for encapsulation is shown in the upper part of the picture, the mortar of the base material is shown in the lower part of the picture, and the coating film is shown in the middle. The coating film was uniformly thick with a thickness of 180 µm.

**Figure 2:**
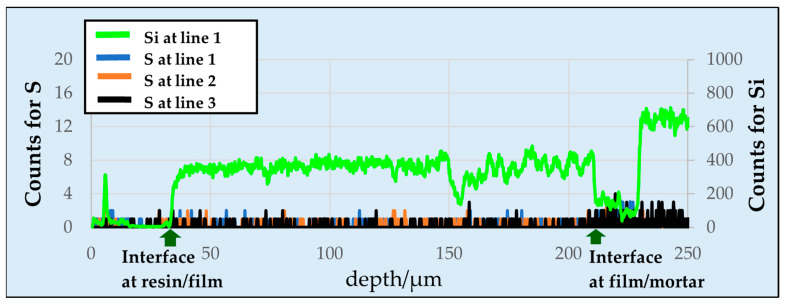
Line analysis of silicon (Si) and sulfur (S) by EPMA. The measured lines are shown in Figure 1.

Figure 2 shows the results of the EPMA line analysis, which revealed that no sulfur was detected in the coating film. As a result, sulfate ions barely diffused through the coating film. Notably, cement was present in the base material’s mortar, and a trace amount of sulfur was detected on the base material side owing to the sulfate ions in the cement.

### 3.2. Polarization Experiment of Polysiloxane Film Formed on Carbon Steel

**Figure 3:**
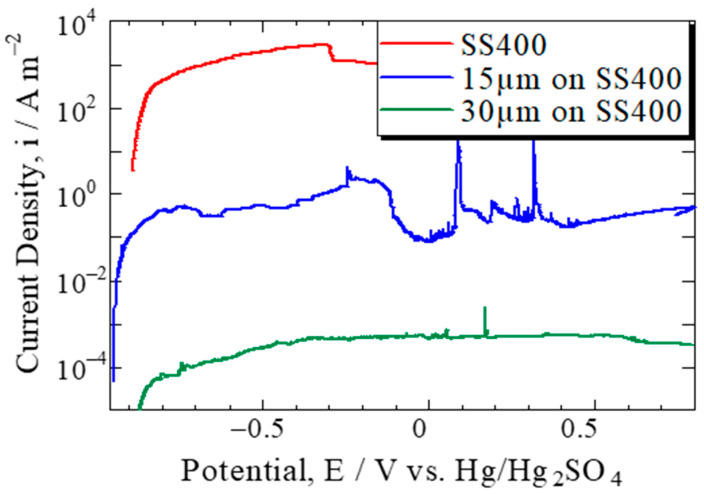
Polarization curves of polysiloxane film with 0, 15, or 30 µm thickness formed on the carbon steel plate.

Figure 3 shows the polarization curves of polysiloxane films with thicknesses of 0, 15, or 30 µm formed on a carbon steel plate. The active current of corrosion of carbon steel was observed between approximately −0.3 and −0.9 V, as indicated in the curve for carbon steel plate without polysiloxane coating, and the passive retention current was observed above approximately −0.3 V. The flame potential was approximately −0.3 V. Table 1 shows a straightforward evaluation of sulfuric acid permeation through the films based on corrosion current at an active state ranging from −0.6 to −0.4 V. Table 1 shows that as the layer thickness increased by 15 µm, the corrosion current decreased by a factor of approximately 2500–3000. As a result, the polarization curves can be used to measure the coating effect quantitatively.

### 3.3. Biofilm Formation Experiment on Polysiloxane Film

**Figure 4:**
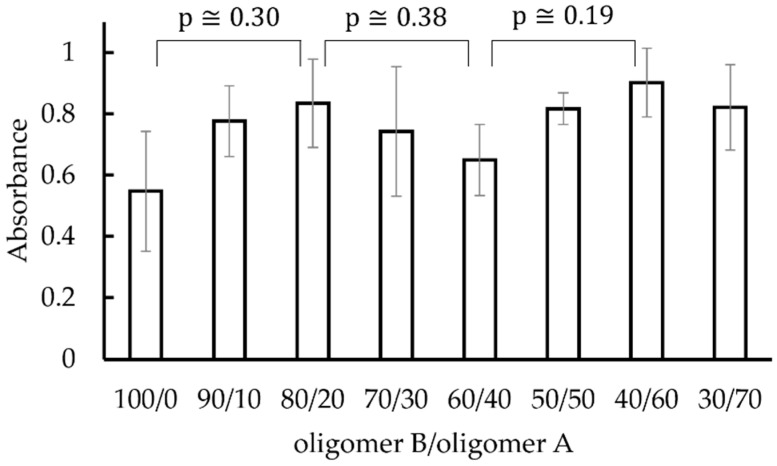
Absorbance of pure water where the stained biofilm, which is formed on polysiloxane films with various compositions of oligomer B/oligomer A ranging from 100/0 to 30/70, was dissolved. The standard error is shown as an error bar.

Figure 4 shows the absorbance of pure water where the stained biofilms, which are formed on polysiloxane films with oligomer B/oligomer A compositions ranging from 100/0 to 30/70, were dissolved. The standard error is shown as an error bar. The figure shows that the amount of biofilm formation varies depending on the oligomer B/oligomer A composition ratio. Although the results obtained were not sufficiently significant, the amount of biofilm formation tended to be lower when the oligomer B/oligomer A composition ratio was 100/0 or 60/40. As ethanol reacts with the film when used as the solvent, pure water was used as a solvent for extracting crystal violet in this study. The use of pure water instead of ethanol as the solvent for extracting crystal violet can be one of the reasons for the greater variability than in biofilm quantification by crystal violet staining in previous studies [12,13].

### 3.4. Wettability Experiment of Pure Water and Simulated Biofilm Droplet on Polysiloxane Film

**Figure 5:**
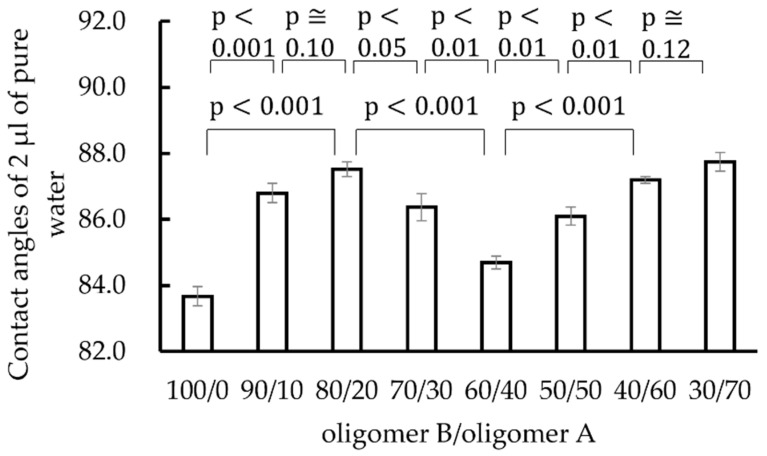
Contact angles of 2 µL of pure water droplets on polysiloxane films with various compositions of oligomer B/oligomer A ranging from 100/0 to 30/70. The standard error is shown as an error bar.

Figure 5 shows the contact angles of 2 µL of pure water droplets on polysiloxane films with various compositions of oligomer B/oligomer A ranging from 100/0 to 30/70. The standard error is shown as an error bar. The figure shows that there is a difference in contact angles depending on the composition ratio of oligomer B/oligomer A. In particular, when the composition ratio of oligomer B/oligomer A was 100/0 or 60/40, the contact angle was smaller than for the other composition ratios.

**Figure 6:**
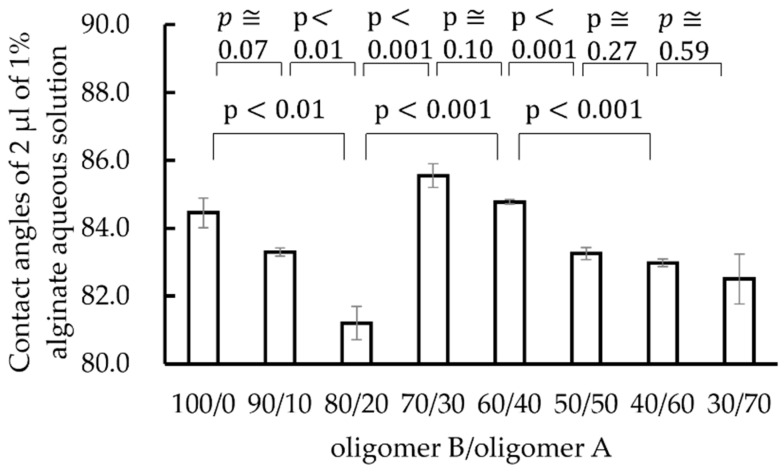
Contact angles of 2 µL of 1% alginate aqueous solution droplets on polysiloxane films with various compositions of oligomer B/oligomer A ranging from 100/0 to 30/70. The standard error is shown as an error bar.

Figure 6 shows the contact angles of 2 µL of 1% alginate aqueous solution droplets on polysiloxane films with various oligomer B/oligomer A compositions ranging from 100/0 to 30/70. The standard error is shown as an error bar. The figure shows that contact angles differ depending on the oligomer B/oligomer A composition ratio. The contact angle was higher when the oligomer B/oligomer A composition ratio was 100/0 or 70/30 (60/40).

**Figure 7:**
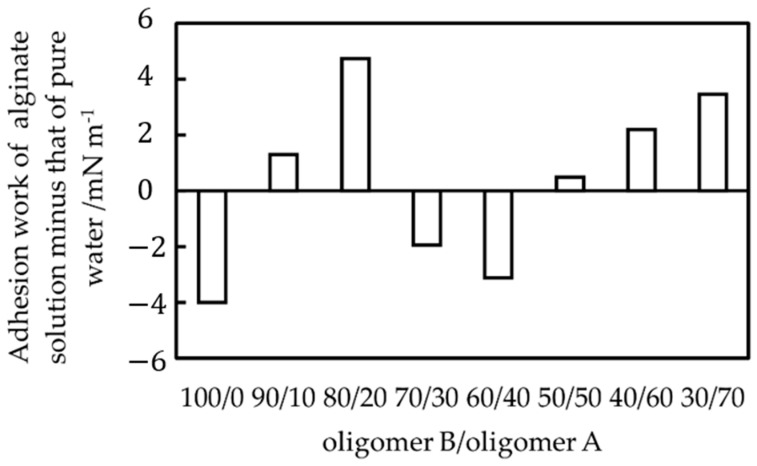
Effect of substrate species on the adhesion work of an alginate solution minus the adhesion work of pure water.

Figure 4, Figure 5 and Figure 6 are comparable. The substrate having a smaller contact angle with pure water and a larger contact angle with a biofilm-simulating alginate aqueous solution is less likely to form a biofilm. The causes of the above tendency were discussed from the perspective of adhesion work. Antimicrobial qualities can influence resistance to biofilm formation in water, but because all the films in this study were polysiloxane films, no substantial difference in antimicrobial properties would be observed. For example, a study [14] showed that when *Pseudomonas aeruginosa* biofilms were generated on a silver substrate with antimicrobial properties versus a tin substrate without antimicrobial properties, the former produced more biofilms than the latter. The authors focused on biofilm adhesion work as one of the influences on the resistance to biofilm development other than antibacterial capabilities. Biofilms are known to have some barrier effect against the outside world [15], and the authors reasoned that whether a biofilm spreads easily on the substrate affects the biofilm’s development behavior. From the experimental results, we calculated the adhesion work of pure water and that of 1% alginate solution as a simulated biofilm to each substrate and evaluated whether the value of the former minus the latter was related to the difficulty of biofilm adhesion. Figure 7 shows the effect of substrate species on the adhesion work of an alginate solution minus the adhesion work of pure water. In Figure 4, the substrate with less biofilm development tended to have a positive value in Figure 7, supporting the hypothesis that the difference in adhesion work between pure water and simulated biofilm is related to the difficulty in biofilm formation. The greater variation in Figure 4 and the smaller variation in Figure 5 and Figure 6 suggest that the prediction of biofilm adhesion behavior from adhesion work of an alginate solution and pure water are adequate. Other effects, such as the structure and unevenness of the substrate, as well as the interfacial energy between water and biofilm, are known to affect biofilm adhesion difficulty in general. In this experiment, all substrates were formed by specifying a mixture of oligomer A and oligomer B. Since the difference in other effects owing to the difference in substrate type was small, we believe that these effects were not noticeable.

## 4. Conclusions

In this study, the sulfuric acid permeation behavior and biofilm formation behavior of polysiloxane films have been investigated, and simple evaluation methods for the sulfuric acid permeation and biofilm formation behaviors have been proposed. The results obtained are as follows:

(1) The polysiloxane films used in these experiments are almost impermeable to the sulfuric acid aqueous solution, and the amount of biofilm formation varies with the film composition.

(2) The amount of sulfuric acid permeation can be estimated by measuring the polarization curves of polysiloxane films with different thicknesses formed on iron electrodes.

(3) We can estimate the resistance of biofilm formation on the polysiloxane films by measuring the adhesion work of pure water droplets and simulated biofilm droplets on polysiloxane films of different compositions.

## Figures and Tables

**Table 1:** Simple evaluation of sulfuric acid permeation through the films estimated from the corrosion current at active state from −0.6 to −0.4 V.

	At −0.6V	At −0.5V	At −0.4V
SS400	1.4 × 10^3^	1.9 × 10^3^	2.4 × 10^3^
15 µm on SS400	4.2 × 10^−1^	5.5 × 10^−1^	5.4 × 10^−1^
30 µm on SS400	1.6 × 10^−4^	2.9 × 10^−4^	4.0 × 10^−4^
